# Supplementary material for: A Comprehensive Assessment of Ultraviolet-Radiation-Induced Mutations in Flammulina filiformis Using Whole-Genome Resequencing
Source: J Fungi (Basel). 2024 Mar 20;10(3):228. doi: 10.3390/jof10030228 (PMC10971301; doi:10.3390/jof10030228)
Supplement: Supplementary file 1 [file jof-10-00228-s001.zip › Supplementary Material S8/KEGG annotation/out/64381550635650.os/KO/out_map/map04660.html]

KEGG PATHWAY: T cell receptor signaling pathway - Reference pathway


|  |  |
| --- | --- |
| **T cell receptor signaling pathway - Reference pathway** |  |

[
Pathway menu
| Organism menu
| Pathway entry
| Show description
| User data mapping
]

Activation of T lymphocytes is a key event for an efficient response of the immune system. It requires the involvement of the T-cell receptor (TCR) as well as costimulatory molecules such as CD28. Engagement of these receptors through the interaction with a foreign antigen associated with major histocompatibility complex molecules and CD28 counter-receptors B7.1/B7.2, respectively, results in a series of signaling cascades. These cascades comprise an array of protein-tyrosine kinases, phosphatases, GTP-binding proteins and adaptor proteins that regulate generic and specialised functions, leading to T-cell proliferation, cytokine production and differentiation into effector cells.

|  |  |
| --- | --- |
| Reference pathway | 100% |
